# Enhanced Oxygen Vacancies in a Two-Dimensional MnAl-Layered Double Oxide Prepared via Flash Nanoprecipitation Offers High Selective Catalytic Reduction of NO*_x_* with NH_3_

**DOI:** 10.3390/nano8080620

**Published:** 2018-08-15

**Authors:** Dan Zhao, Chao Wang, Feng Yu, Yulin Shi, Peng Cao, Jianming Dan, Kai Chen, Yin Lv, Xuhong Guo, Bin Dai

**Affiliations:** 1 Key Laboratory for Green Processing of Chemical Engineering of Xinjiang Bingtuan, School of Chemistry and Chemical Engineering, Shihezi University, Shihezi 832003, China; zhaod2015@163.com (D.Z.); wangchao_shzu@163.com (C.W.); shiyulin521@126.com (Y.S.); caop@shzu.edu.cn (P.C.); djm_tea@shzu.edu.cn (J.D.); chenkai@shzu.edu.cn (K.C.); ag_125@163.com (Y.L.); 2 State Key Laboratory of Chemical Engineering, School of Chemical Engineering, East China University of Science and Technology, Shanghai 200237, China

**Keywords:** layered double oxide, oxygen vacancies, selective catalytic reduction, low-temperature denitration, flash nanoprecipitation

## Abstract

A two-dimensional MnAl-layered double oxide (LDO) was obtained by flash nanoprecipitation method (FNP) and used for the selective catalytic reduction of NO*_x_* with NH_3_. The MnAl-LDO (FNP) catalyst formed a particle size of 114.9 nm. Further characterization exhibited rich oxygen vacancies and strong redox property to promote the catalytic activity at low temperature. The MnAl-LDO (FNP) catalyst performed excellent NO conversion above 80% at the temperature range of 100–400 °C, and N_2_ selectivity above 90% below 200 °C, with a gas hourly space velocity (GHSV) of 60,000 h^−1^, and a NO concentration of 500 ppm. The maximum NO conversion is 100% at 200 °C; when the temperature in 150–250 °C, the NO conversion can also reach 95%. The remarkable low-temperature catalytic performance of the MnAl-LDO (FNP) catalyst presented potential applications for controlling NO emissions on the account of the presentation of oxygen vacancies.

## 1. Introduction

Nitrogen oxides (NO*_x_*) emitted from automobile exhaust gas and the industrial combustion of fossil fuels are air pollutants [1,2,3]. The selective catalytic reduction (SCR) of NO*_x_* with NH_3_ is currently the most effective method to control NO*_x_* emissions [4]. Supported Mn-based catalysts have attracted widespread attention because of the large amount of free oxygen on their surface—this plays a key role in the field of low-temperature SCR reactions. There are many choices of supports for Mn-based catalysts at low temperature, including Mn/zeolite [5]. Mn/activated carbon [6,7], Mn/TiO_2_ [8,9], Mn/Al_2_O_3_ [10], Mn/ZrO_2_ [11], and Mn/Vermiculite (VMT) [12]. In general, a suitable catalyst carrier should have unique properties, including strong surface acids, a high specific surface area, high thermal stability, and strong mechanical strength [13].

Mn-based mixed metal oxides (MMOs) have also attracted much attention. Studies of metal oxides as SCR catalysts have suggested that carrier-free metal oxides still offered good denitrification performance, due to the interactions among the spin, charge, and lattice. Examples of these catalysts include (Fe) MnTi [14], (Cu, Mn)-Mg-Al [15], and Cu_2_Mn_0.5_Al_0.5_O*_x_* [10]. Many reports have described the fine preparation processes to control the morphology, structure, and even the crystal configuration of the catalysts. Novel synthetic methods include co-precipitation [16], hydrothermal procedure [17], the supercritical antisolvent process [18], ion-exchange wet impregnation [19], spray drying [20], and self-propagating high-temperature synthesis [21].

Two-dimensional layered double hydroxide (LDH)-derived MMOs have recently attracted widespread attention because of their special two-dimensional structures, higher active sites, and specific surface areas [22,23,24]. For example, two-dimensional (2D) Co-Al MMOs that are derived from LDH have been investigated as a potential catalyst for NO*_x_* removal at high temperatures of 750 °C in the presence of oxygen and water [25]. Furthermore, a 2D layered Cu-based catalyst from LDH has been used as an SCR catalyst [26]. Mrad et al. [27] reported the SCR of NO by C_3_H_6_ over a series of hydrotalcite-based Cu-Mg-Al-Fe MMO catalysts prepared via co-precipitation. Zhang et al. [28] synthesized Cu-Zn-Al MMO catalysts derived from LDHs, and reported a NO*_x_* conversion of 80% at 240 °C. Yan et al. [29] prepared highly dispersed Cu-Al MMO—the NO*_x_* conversion of Cu-Al MMOs was as high as 84.7%, which is much higher than that of the control catalyst 10 wt % CuO/gamma-Al_2_O_3_ (57.5%). Manganese doping in LDH has also been studied. Yan et al. [10] prepared LDH-derived Cu_2_Mn_0.5_Al_0.5_O*_x_* that resulted in a NO*_x_* conversion of 91.2% at 150 °C, which is much higher than that of all other control catalysts: Cu_2_AlOx (71.1%), Cu-Mn/gamma-Al_2_O_3_ (65.23%), and Mn/gamma-Al_2_O_3_ (59.32%).

In this work, we designed and prepared two-dimensional (2D) MnAl-layered double oxide (LDO) with many oxygen vacancies via flash nanoprecipitation method (FNP). These MnAl-LDO catalysts offered improved low-temperature catalytic activities due to their unique thin layer structures and rich surface oxygen concentrations. Moreover, the FNP method can quickly prepare samples [30]. Metal oxides rapidly crystallize and cause lattice distortion and charge compensation—this leads to oxygen vacancies. Therefore, the FNP method provides a simple strategy for the preparation of environmentally-friendly catalysts with a layered structure. The 2D MnAl-LDO (FNP) catalysts exhibited a 2D layered structure, resulting in enhanced active sites. This led to excellent low-temperature performance in an SCR reaction at 50–250 °C. This FNP method can prepare catalysts with high levels of oxygen vacancies.

## 2. Materials and Methods

### 2.1. Catalyst Preparation

The traditional coprecipitation (CP) preparation of Mn-Al hydrotalcite-like compounds used Mn(CH_3_COO)_2_·4H_2_O as the Mn source and Al (NO_3_)_3_·9H_2_O as the Al source dissolved into distilled water to obtain the metal precursor solutions. Here, the Mn:Al molar ratio was 1:1, and the content of Mn was about 29 wt %. Next, a 1 mol/L NaOH solution was slowly added as the precipitant until the samples were pH = 9. The resulting slurry was aged at room temperature for 8 h and washed with deionized water until the pH reached neutrality; it was then dried at 80 °C. This sample was named MnAl-LDH (CP). The hydrotalcite precursor was then calcined at 550 °C in air for 6 h and it was termed MnAl-LDO (CP).

**Figure 1:**
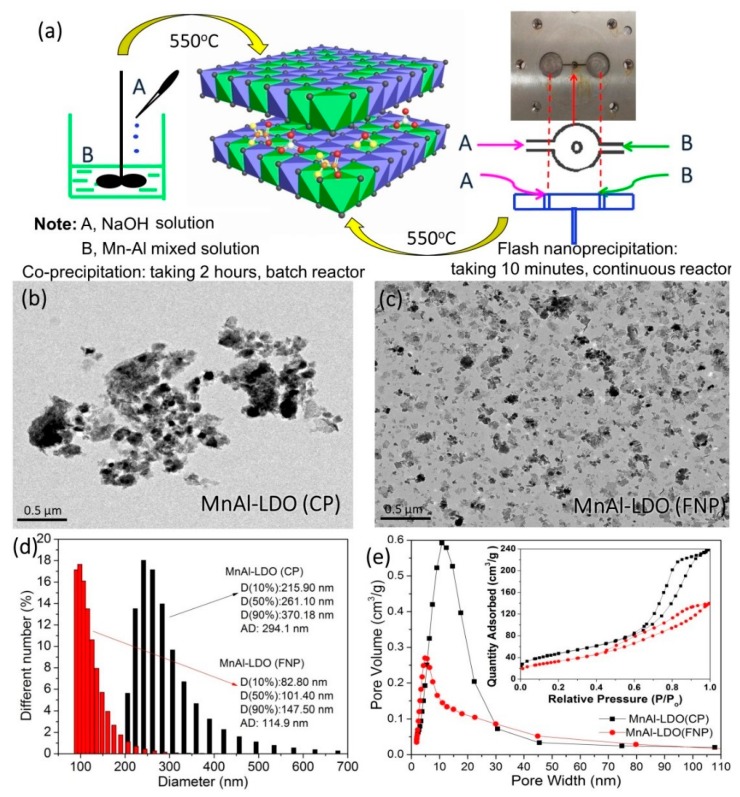
(**a**) The preparation schematic diagram; (**b**,**c**) Transmission electron microscopy (TEM) images; (**d**) Pore size distribution curves; (**e**) N_2_ adsorption-desorption isotherms and pore size distributions of MnAl-LDO (MnAl-layered double oxide) (CP) and MnAl-LDO (FNP) catalysts.

The FNP approach also used Mn(CH_3_COO)_2_·4H_2_O and Al (NO_3_)_3_·9H_2_O, dissolved into distilled water to obtain the metal precursor solutions (solution A); a 1 mol/L NaOH precipitant served as solution B. Solutions A and B were injected into the impinging stream reactor via the injection needle of the FNP equipment [31,32]. The liquid flow rate was adjusted over several iterations to control the pH of the product at 7. The resulting slurry was aged at room temperature for 8 h, and washed with deionized water until the pH was neutral. The samples were then dried at 80 °C. The product was named MnAl-LDH (FNP). This hydrotalcite precursor was calcined at 550 °C in air for 6 h and termed MnAl-LDO (FNP). The preparation schematic diagram is shown in Figure 1a.

### 2.2. Material Characterization

The specific surface area and pore size distribution of the catalyst were measured by a US ASAP2020C (Micromeritics Instrument Co., Norcross, GA, USA) N_2_ adsorption-desorption instrument. The catalyst was first vacuum degassed at 200 °C, cooled to room temperature, and then subjected to N_2_ desorption experiments at −196 °C. The particle size distribution was measured by a laser particle analyzer (NanoPlus-3, NanoPlus, Gerbrunn, Germany). X-ray diffraction (XRD) was used to characterize the crystal form of the catalyst metal oxide. The instrument model was a Bruker D8 ADVANCE (Bruker Biosciences Co., Billerica, MA, USA), and the ray source was Cu-Kα. The test conditions were at 40 mV and 40 mA, with a scanning range of 10–90°. H_2_ temperature programmed reduction (H_2_-TPR) (temperature-programmed reduction) was used to determine the reduction performance of the catalyst. The instrument used was Micrometic ASAP 2720 Multisorbent (Micromeritics Instrument Ltd., Norcross, GA, USA). The test conditions were as follows: the catalyst was purged with nitrogen for 20 min at 100 °C, and then it was fed with a 10% H_2_/Ar gas mixture at a flow rate of 38 mL/min. The thermal conductivity detector automatically collected data from room temperature to 650 °C. The transmission electron microscopy (TEM) using a JEOL-2010F electron microscope (JEOL, Tokyo, Japan), operated at 200 kV. We used X-ray photoelectron spectroscopy (XPS) to analyze the electronic states and the atom amounts on the sample surfaces using a Kratos AXIS Ultra DLD (Kratos Analytical Inc., Manchester, UK). The binding energy of C 1 s of 284.8 eV was taken as a reference to the binding energies of the spectra.

### 2.3. Activity Measurement

A micro-structured fixed bed reactor evaluates the catalytic performance of the powdered catalysts in the NH_3_-SCR reaction at the lab-scale. The NH_3_-SCR catalytic activity tests of the synthesized catalysts were carried out at atmospheric pressure in a fixed-bed stainless steel reactor with an internal diameter of 10 mm. The stainless steel reactor was installed in a vertical split-tube furnace. For each test, 0.1 g catalyst was charged. A typical composition of the simulated flue gas was as follows: 500 ppm NH_3_, 500 ppm NO, 5 vol % O_2_, N_2_ as the balance gas, and the total volume flow was 100 mL/min. Its SCR reaction performance was evaluated at different temperatures under flowing simulated flue gas after a 30 min initialization period. The initial NO concentration in the simulated flue gas was 500 ppm, and this was referred to as [NO]_in_. The output gases were evaluated every 50 °C from 50 to 400 °C by Fourier transform infrared spectroscopy (FTIR) (Thermofisher IS10, Thermo Fisher Scientific, Waltham, MA, USA), and the NO content was [NO]_out_. The NO conversion was calculated from the following equations:

(1) $$\mathrm{NO}\;\mathrm{conversion}=\frac{{\left[\mathrm{NO}\right]}_{\mathrm{in}}-{\left[\mathrm{NO}\right]}_{\mathrm{out}}}{{\left[\mathrm{NO}\right]}_{\mathrm{in}}}\times 100\%$$

The N_2_ selectivities were calculated according to the input concentrations of NO*_x_* and NH_3_, and the output concentrations of N_2_O, NO_2_, NO*_x_*, and NH_3_ as follows:

(2) $$S\left(N_{2}\right)=\left[1-\frac{2{\left[N_{2}O\right]}_{\mathrm{out}}+{\left[{\mathrm{NO}}_{2}\right]}_{\mathrm{out}}}{{\left[\mathrm{NO}\right]}_{\mathrm{in}}+{\left[{\mathrm{NH}}_{3}\right]}_{\mathrm{in}}-{\left[\mathrm{NO}\right]}_{\mathrm{out}}-{\left[{\mathrm{NH}}_{3}\right]}_{\mathrm{out}}}\right]\times 100\%$$

## 3. Results and Discussion

Transmission electron microscopy (TEM) images of the samples are shown in Figure 1b,c. These data show that the MnAl-LDO (FNP) catalyst particles were uniform and of micro-diameter distribution. Figure 1d shows that the average particle diameter of the as-obtained catalyst was smaller (114.9 nm) than MnAl-LDO (CP) catalyst (294.1 nm)—this was because of fast settling during preparation. Figure 1e shows the N_2_ adsorption-desorption isotherms and the Barrett-Joyner-Halenda (BJH) pore size distribution curves of MnAl-LDO (CP) and the MnAl-LDO (FNP) catalysts. The adsorption isotherms have a well-defined H_2_-type hysteresis loop at a *p*/*p*_0_ close to saturation—this is characteristic of the plateau/inflection of the mesoporous materials. The pore size distribution of the samples showed two sharp and obvious peaks that were located at 13 nm and 7 nm for MnAl-LDO (CP) and MnAl-LDO (FNP) catalysts, respectively [33]. The Brunauer-Emmett-Teller (BET) surface area, pore size, and pore volume are slightly reduced, as shown in Table 1.

**Figure 2:**
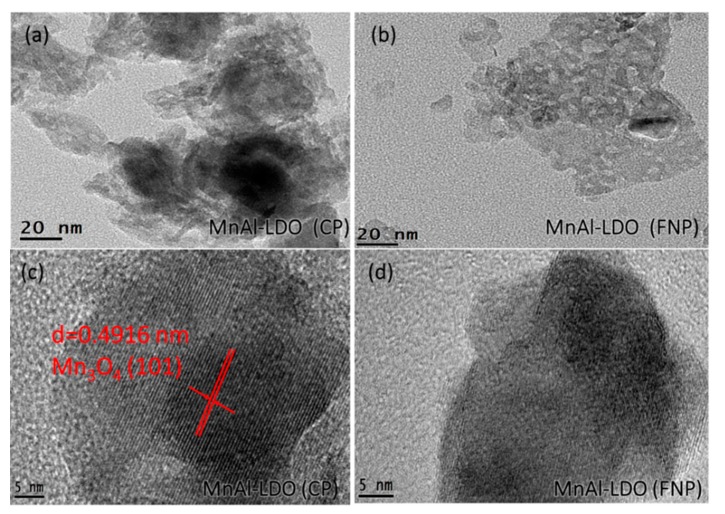
HR-TEM images of MnAl-LDO (CP) and MnAl-LDO (FNP) catalysts.

The high resolution TEM (HR-TEM) images of these catalysts (Figure 2a,b) showed abundant irregular spots on the surface of the MnAl-LDO (FNP) catalyst. This was the pore space. The MnAl-LDO (CP) catalyst had an obvious black shadow because of slight agglomeration. To study particle composition, HR-TEM images with a 5 nm gauge are shown in Figure 2c,d. The MnAl-LDO (CP) catalyst had well-defined lattice fringes with a d-spacing about 0.49 nm corresponding to the (101) plane of Mn_3_O_4_. However, the MnAl-LDO (FNP) catalyst has a d-spacing of the lattice fringes of about 0.25 nm. This uniform distribution of nanoparticles might be because of the high-speed collisions during FNP, which stopped the growth cycle of the crystals.

**Figure 3:**
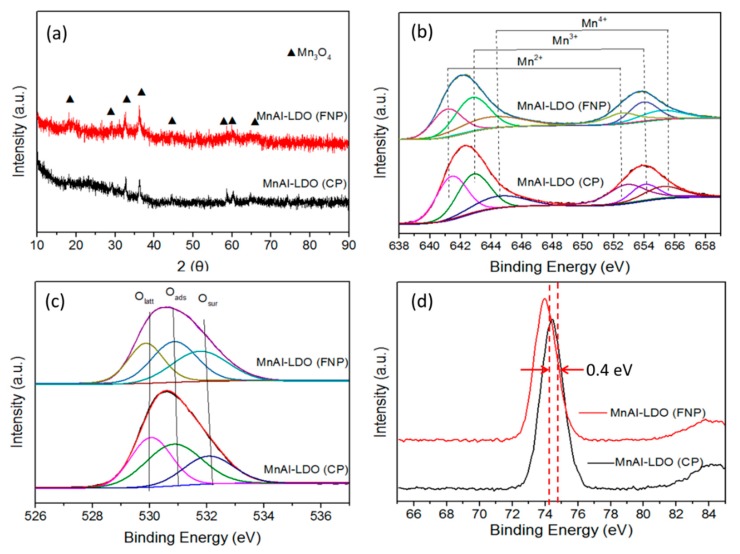
(**a**) XRD patterns of the MnAl-LDO (CP) and MnAl-LDO (FNP) catalysts; (**b**) Mn 2p; (**c**) O 1s and (**d**) Al 2p XPS spectra of the MnAl-LDO (CP) and MnAl-LDO (FNP) catalysts.

XRD helped to clarify the crystal phases of the MnAl-LDO (CP) and MnAl-LDO (FNP) catalysts (Figure 3a). The diffraction line profile of the MnAl-LDO (CP) catalyst showed diffraction peaks at 18.0°, 28.9°, 32.4°, 60.0°, 43.6°, 58.7°, 60.0°, and 64.6°, attributed to Mn_3_O_4_, as predicted by PDF#18-0803 [12]. In addition, there is no evidence of an XRD-detectable Al-related oxide phase in any of the catalysts, which suggests an amorphous character for alumina, and/or an Al-bearing mixed oxide phase.

The elementary oxidation states and surface compositions of the samples were investigated by XPS. Figure 3b shows that the Mn 2p spectra of the samples were fitted with the Mn 2p_3/2_ and Mn 2p_1/2_ of MnO*_x_*. Previous research has shown that peaks at 642.7 eV and 653.4 eV were Mn^3+^ from Mn_2_O_3_, the peaks at 644.3 eV and 655.1 eV were Mn^4+^ of MnO_2_, and the peaks at 641.5 eV and 652.3 eV were attributed to Mn^2+^. As shown in Table 2, The Mn^3+^ content of both samples was similar, but the Mn^4+^/Mn ratio in MnAl-LDO (FNP) was higher than in MnAl-LDO (CP). Previous reports proved that a large amount of Mn^4+^ improved NH_3_ adsorption, and the strong interaction between Mn^3+^ and Mn^4+^ amplified catalytic activity in the SCR reaction at low temperature. Exposure to Mn ions could increase oxidation capacity, and provide more reactive oxygen species.

Figure 3c shows the O 1s XPS spectra of MnAl-LDO (CP) and MnAl-LDO (FNP) catalysts. The O 1s spectra of both catalysts were fitted into three peaks: The peak at 530.9 eV corresponded to lattice oxygen, the peak at 531.4 eV was related to the adsorbed oxygen, and the peak at 532.4 eV was attributed to the surface oxygen. Previous reports have shown that in low-temperature SCR reactions, free NH_3_ is absorbed on the lattice oxygen and then activated into an amino group. This generates intermediate products [34,35]. Therefore, the increased lattice oxygen content could enhance the oxidation capacity on the surface of the catalyst and promote a “fast SCR” reaction. Here, the relative lattice oxygen concentration of MnAl-LDO (CP) and MnAl-LDO (FNP) catalysts were 30.42% and 35.44%, respectively (Table 2). Similarly, adsorbed oxygen could be converted to lattice oxygen, and the enhanced adsorbed oxygen content could accelerate the SCR reaction cycle. This would improve the catalytic activity, and the O_ads_ could promote oxidation of NO into NO_2_, and consequently facilitating the “fast SCR” reaction [10].

During the formation of composite metal oxides, Al_2_O_3_ supported a large number of active sites and it improved the specific surface area. Figure 3d shows that the Al 2p diffraction peak of the MnAl-LDO (FNP) catalyst shifted to lower binding energies compared with the MnAl-LDO (CP) catalyst. Previous reports have suggested that the binding energies decreased, due to the interactions with transition metals. These interactions enhanced the number of oxygen vacancies, and when forming a MnAl layered double oxide, the lattice distortion between Mn and Al could promote formation of oxygen vacancies [36,37].

Catalyst surfaces with oxygen vacancies are more active and they promote adsorption near the oxygen vacancies on the surface [38]. Metal vacancies can also tune the electronic structure of the surface and improve the catalytic activity, because of their electron and orbital distributions. The presence of metal vacancies in the catalyst reaction increased the valence state of nearby metal centers [39,40]. When an oxygen vacancy was formed in a metal oxide, the two electrons were singled out, and they could be transferred to neighboring metal ions, reducing the valence of the metal ions [41]. So, the MnAl-LDO (FNP) catalyst had better NO conversion. 

**Figure 4:**
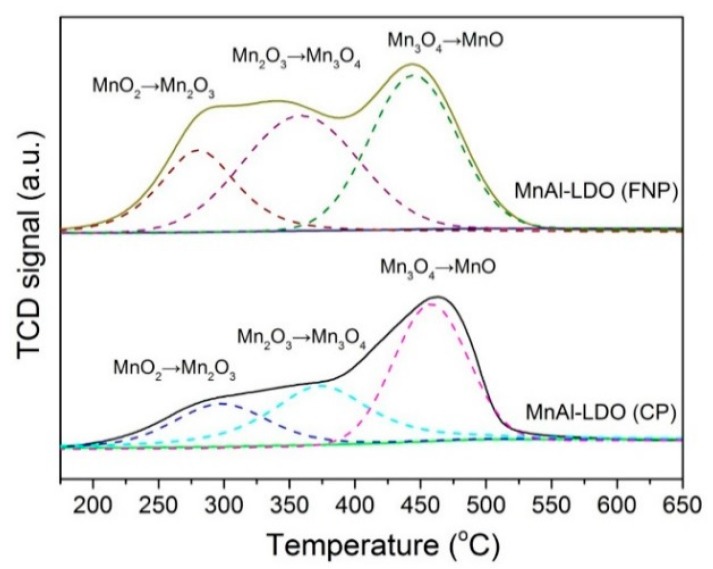
H_2_-TPR profiles of MnAl-LDO (CP) and MnAl-LDO (FNP) catalysts.

The surface redox property was a key characteristic of SCR catalysts. Figure 4 shows the H_2_-TPR profiles of MnAl-LDO (CP) and MnAl-LDO (FNP) catalysts. The reduction curves of the MnAl-LDO (CP) catalyst showed a distinct reduction peak at 180–550 °C. This could be fitted with three peaks: the peak at 280 °C represented MnO_2_ to Mn_2_O_3_, the peak at 349 °C represented Mn_2_O_3_ to Mn_3_O_4_, and the peak at 450 °C was the further reduction of Mn_3_O_4_ to MnO, agreeing well with the results of XPS analysis [42,43]. Generally, the curves corresponded to the amount hydrogen uptake over the catalysts, and the peak locations mean how easy the catalyst species are reduced. Moreover, the peak area in the profile of MnAl-LDO (FNP) catalyst was larger than that in the profile of MnAl-LDO (CP) catalyst. The enhancement was mainly due to the synergistic effect between the Mn and Al, which can create oxygen defects and oxygen vacancies, which suggested that the mobility of surface oxygen was enhanced and beneficial to the SCR reaction [44]. Meanwhile, the strong reduction performance of MnAl-LDO (FNP) catalyst offered richer active sites on the surface. All these features indicated that the modification with the new method and rich oxygen vacancies could greatly increase the redox ability of the MnAl-LDO (FNP) catalyst, which was favorable to enhance its SCR activity.

**Figure 5:**
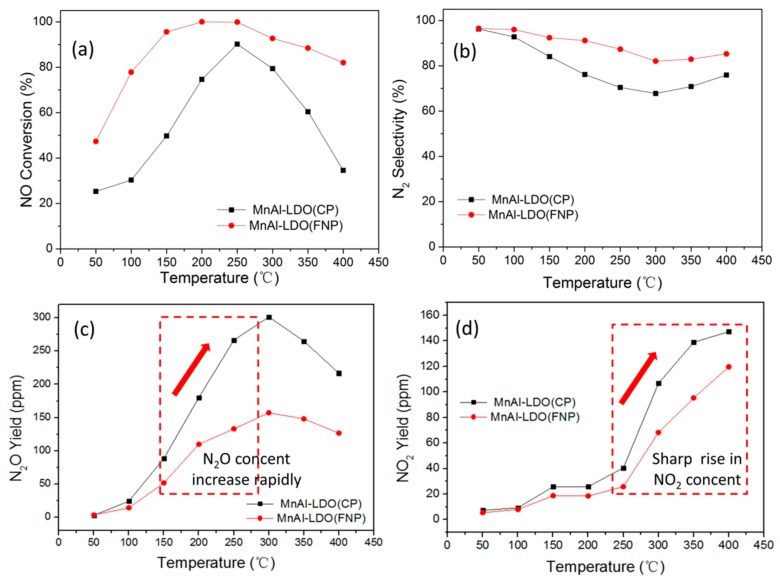
(**a**) NO conversion; (**b**) N_2_ selectivity; (**c**) N_2_O yield and (**d**) NO_2_ yield of MnAl-LDO (CP) and MnAl-LDO (FNP) catalysts (GHSV: 60,000 h^−1^).

The catalytic performance of both catalysts at a GHSV of 60,000 h^−1^ is shown in Figure 5. The NO conversion of MnAl-LDO (FNP) was 48% at 50 °C (MnAl-LDO (CP) was only 24%). At 250 °C, the NO conversions of both catalysts increased gradually and reached a maximum at 250 °C, and then they decreased with increasing temperature. The MnAl-LDO (FNP) catalyst had a broad temperature window of 100–400 °C, in which NO conversion was over 80%. This window was only 200–300 °C for MnAl-LDO (CP). Table 3 details a two-dimensional MnAl-layered double oxide prepared via FNP. It had remarkable NO conversion. Figure 5b shows the N_2_ selectivity; when the temperature was below 250 °C, the N_2_ selectivity of MnAl-LDO (FNP) catalysts maintained 90%, and it then it sharply decreased, because of the production of NO_2_ and N_2_O. The MnAl-LDO (CP) catalysts had N_2_ selectivity that rapidly decreased above 100 °C, due to the high amounts of byproducts, because of the lower production of N_2_O and NO_2_. Figure 5c shows the N_2_O yield. The curve was on the upward trend, when the temperature was between 50–300 °C, the N_2_O generation increases with fastest rate, when the temperature between 300–400 °C, the N_2_O generation decreases. Figure 5d shows the NO_2_ yield; when the temperature between 50–250 °C, the curve leveled off, NO_2_ formation changed a little, and were the lowest at 200 °C. When the temperature was between 250–400 °C, the curve was on the rise, and going up very fast, reaching the highest at 400 °C. 250 °C was equivalent to a cut-off point; the temperature was less than 250 °C, and the NO_2_ generation was very low; when the temperature was higher than 250 °C, the NO_2_ generation increased significantly.

Before the SCR reaction, the analog flue gas had no NO_2_ and N_2_O. However, the output gas contained numerous byproducts with increasing concentrations. The N_2_O concentration rose sharply above 150 °C due to the following reaction: 4NO + 4NH_3_ + 3O_2_ → 4N_2_O + 6H_2_O [45]. Of note, the N_2_O concentration gradually decreased, due to the strong reduction property of NH_3_ at high temperature. At temperatures over 250 °C, the concentration of NO_2_ increased dramatically, and this could lead to the following: 2NO + O_2_ → 2NO_2_ and 2NH_3_ + 7O_2_ → 2NO_2_ + 3H_2_O [46]. In the presence of oxygen, NO is oxidized to NO_2_, and then NO_2_ is reduced in the presence of a reductant to N_2_, and the reaction rate would be increased if NO_2_ was generated in the reaction system. This corresponds to the fast-SCR reaction shown in the equation. 4NH_3_ + 2NO + 2NO_2_ → 4N_2_ + 6H_2_O [47]. Furthermore, the content of NO*_x_* (NO + NO_2_ + N_2_O) was higher than the original NO concentration (500 ppm) at 350 °C, which suggested that the NH_3_ nitrogen source was oxidized to form the NO or NO_2_ at high temperature, as shown in Figure 5c,d.

## 4. Conclusions

The MnAl-LDO successfully prepared by a co-precipitation method and a flash nanoprecipitation method, as an excellent NH_3_-SCR catalyst. XRD, XPS, and TPR analyses demonstrated that a series of Mn-Al hydrotalcite were good precursor catalysts. The best catalysts, such as MnAl-LDO (FNP), showed much higher catalytic activity than the MnAl-LDO (CP) catalyst. The maximum NO conversions is 100% at 200 °C, and it had a narrow activity temperature window of 150–300 °C (NO conversion > 90%). MnAl-LDO (CP) exhibits more inferior NO conversion, and the highest conversion of NO is only 74.58% at 200 °C. MnO_2_ is the main active components. In addition, the high concentrations of Mn^4+^ should also be partly responsible for the good performance of the MnAl-LDO (FNP) catalyst, with rich oxygen vacancies also being beneficial to NO conversion. H_2_-TPR analyses indicated that MnAl-LDO (FNP) possesses a higher reducibility than the MnAl-LDO (CP) catalysts.

## Figures and Tables

**Table 1:** The BET surface area, pore volume, and pore size of MnAl-LDO (CP) and MnAl-LDO (FNP) catalysts.

Samples	BET Surface Area (m^2^/g)	Pore Volume (cm^3^/g)	Pore Size (nm)
MnAl-LDO (CP)	169	0.37	8.77
MnAl-LDO (FNP)	121	0.22	7.13

**Table 2:** Surface atomic concentration of various elements in MnAl-LDO (CP) and MnAl-LDO (FNP) catalysts.

Samples	Surface Atomic Concentration (%)								
	Al	Mn	O	Mn^2+^/Mn	Mn^3+^/Mn	Mn^4+^/Mn	O_latt_	O_ads_	O_surf_
MnAl-LDO (CP)	22.88	9.56	57.24	34.9	41.6	23.5	30.42	35.32	34.26
MnAl-LDO (FNP)	21.66	9.28	56.35	31.8	41.4	26.8	35.44	40.57	23.99

Note: O_latt_, O_ads_, O_surf_ stand for lattice oxygen, adsorbed oxygen and surface oxygen, respectively.

**Table 3:** Summary of catalytic activity for various Mn-based catalysts. In the table, GHSV means gas hourly space velocity, while MMO stands for mixed metal oxide.

Mn-Based Catalysts	Synthesis Methods	Temperature (°C)	GHSV (h^−1^)	NO Content (ppm)	Conversion	Ref.
Mn/γ-Al_2_O_3_	Impregnation	200	-	500	NO*_x_*: 67.2%	[14]
Cu-Mn/γ-A_l2_O_3_	Impregnation	200	-	500	NO*_x_*: 82.6%	[14]
Mn-Fe/VMT	Impregnation	200	30,000	500	NO: 96.5%	[12]
Cu_2_Mn_0.5_Al_0.5_Ox	Co-precipitation	150	-	500	NO*_x_*: 91.2%	[10]
Mn-Ce-Al (MMO)	Spray drying	150	15,000	500	NO*_x_*: 97.4%	[20]
Mn–Ce/γ-Al_2_O_3_	Sol-gel	300	30,000	700	NO: 85%	[41]
40 wt %Mn_0.75_Fe_0.25_/Al_2_O_3_	Deposition precipitation	150	-	1000	NO: 71%	[44]
MnO*_x_*-CeO_2_-Al_2_O_3_	Flash-nanoprecipitation	150	15,300	500	NO*_x_*: 90%	[30]
MnAl-LDO (CP)	Co-precipitation	200	60,000	500	NO: 74.68%	This work
MnAl-LDO (FNP)	Flash-nanoprecipitation	200	60,000	500	NO: 100%	This work
